# Application of hospital–community–home linkage management model in patients with type 2 diabetic nephropathy

**DOI:** 10.1186/s41043-024-00521-7

**Published:** 2024-03-07

**Authors:** Hong-Mei Xu, Yan-Ping Zhai, Wen-Juan Zhu, Min Li, Zhi-Ping Wu, Peng Wang, Xue-Jing Wang

**Affiliations:** 1 Department of Education, Shanxi Bethune Hospital, Taiyuan, 030032 Shanxi China; 2 Department of Internal Medicine, Shanxi Bethune Hospital, Xiaodian District, 99 Longcheng Street, Taiyuan, 030032 Shanxi China; 3 Department of Nursing, Shanxi Bethune Hospital, Taiyuan, 030032 Shanxi China; 4 Department of Breast Surgery, Shanxi Bethune Hospital, Taiyuan, 030032 Shanxi China; 5 Department of Nursing, Taiyuan City Central Hospital, Taiyuan, 030009 Shanxi China

**Keywords:** HCH management, Diabetic nephropathy, Nutrition management, Compliance behaviour, Blood glucose and kidney function

## Abstract

**Objective:**

To explore the effect of the hospital–community–home (HCH) linkage management mode in patients with type 2 diabetic nephropathy (DN).

**Method:**

A total of 80 patients with type 2 DN hospitalised in the Department of Nephrology of our hospital between July 2021 and June 2022 were recruited and subsequently divided into the observation group and the control group using the random number table method, with 40 patients in each group. The control group received routine health education and discharge guidance. The HCH linkage management model was implemented for the observation group based on routine care. The improvements in compliance behaviour, biochemical parameters of renal function, blood glucose level and self-management ability were compared before the intervention and at 3 and 6 months after the intervention.

**Results:**

After the intervention, the scores for compliance behaviour of the observation group were better than those of the control group, with a statistically significant difference (*P* < 0.05). The biochemical indicators of renal function and blood glucose level were significantly lower in the observation group compared with in the control group, with a statistically significant difference (*P* < 0.05). After the intervention, the observation group showed a great improvement in self-management ability and cognition of the disease, with significant differences (*P* < 0.05).

**Conclusion:**

The HCH linkage management mode can improve the compliance behaviour of patients with type 2 DN, effectively improve the renal function and blood sugar level of patients, enhance the self-management ability and cognition of the disease and delay the development of the disease.

## Introduction

Diabetic nephropathy (DN) is a progressive decline in glomerular filtration rate (GFR) and proteinuria caused by a long period of diabetes. It is the most common chronic complication of diabetes, with varying degrees of renal changes after 10 years of diabetes [1, 2]. According to statistics, the incidence of type 2 diabetes complicated by nephropathy is 20–60% [3], with a rapid growth trend [4]. Most patients with diabetes have a long course of disease. Due to their lack of cognition of the disease and poor self-management ability, patients can experience rapid kidney involvement and renal complications, which can easily develop into end-stage renal disease. Diabetic nephropathy has become the second cause of end-stage renal disease, and it seriously affects the quality of life of patients [5]. Metabolic disorders in patients with DN, often accompanied by insufficient insulin secretion, insulin resistance and other symptoms, result in the nutritional treatment becoming difficult and complex. When developing into stage III of DN, the treatment should not only ensure that patients consume enough energy and nutrients every day but also control the quality and quantity of carbohydrates, fat, protein and other nutrients to meet the special metabolic needs of the body. However, patients can find it difficult to understand professional and complex nutrition education and dietary guidance, with poor acceptance and compliance. Moreover, nutrition management is a long-term process, and it is difficult for the general supervision and training process to achieve good results. Therefore, the prevention and treatment of DN through nutritional management is both necessary and significant to delay the progression into end-stage renal disease.

Hospital–community–home (HCH) is a hierarchical management, three-level linkage, seamless and two-way circulation management model. The advantageous characteristics of HCH nutrition management are the extension and expansion of the four core issues: nutrition management content, nutrition management scope, nutrition management object and nutrition management purpose. The aim is to improve the capacity of long-term care and health education for chronic diseases [6, 7].

The purpose of this study was to evaluate and analyse the effect of HCH nutritional management for the control of disease progression in patients with type 2 DN. We found that the HCH linkage management model in patients with type 2 DN allowed for continuous treatment and care and effectively controlled the progression of the disease.

## Methods and materials

### Study participants

We conducted a randomized, parallel controlled, single-centre study. A total of 80 patients with type 2 DN hospitalised in the Department of Nephrology between July 2021 and June 2022 were selected and numbered in the order of admission. The patients were randomised into the observation group (*n* = 40) and the control group (*n* = 40) using the random number table method. The observation group received the intervention outlined below, and the control group received routine care for DN including assessment of diseases, health guidance for related diseases, regular health lectures (weekly), guidance for medication, re-diagnosis and lifestyle education. Regular follow-up was conducted by responsible nurses monthly, and online services were provided by Internet if necessary. This study aimed to compare the scores for compliance behaviour, renal function and self-management between the two groups after 1 year of intervention and observation.

The patients met the relevant diagnostic criteria for type 2 DN [8]. The inclusion criteria were as follows: (1) patients with type 2 diabetes mellitus combined with chronic kidney disease where the condition was relatively stable; (2) patients aged > 40 years old; (3) patients clearly aware of and fully coordinated with this project; and (4) patients or family members who provided informed consent and cooperated. The exclusion criteria included: (1) patients with DN who have undergone haemodialysis; (2) patients with other serious medical diseases and poor general condition; and (3) patients who cannot take care of themselves.

This study was conducted in accordance with the Declaration of Helsinki and was approved by the Research Ethics Committee of Shanxi Bethune Hospital (Ethical Batch Number: YXLL-2022-104), with informed consent obtained from all participants. All methods were carried out in accordance with relevant guidelines and regulations.

### Study methods

#### Preintervention preparation

Before the intervention, the patients should be fully evaluated, and the files of patients with DN should be established to collect and determine all relevant indicators according to the doctor’s advice.

#### Construction of the intervention programme

##### Establishment of hospital–community–home management team

The study team consisted of three physicians (two nephrologists, one endocrinologist), six charge nurses and one nutritionist, along with patients with DN and family caregivers. The two nephrologists were responsible for the development of intervention programmes and the training of intervention personnel, while the endocrinologist was responsible for the measurement and adjustment of intervention indicators. One nurse was responsible for the patient file establishment and quality control, with the remaining nurses responsible for the implementation of the intervention programme. The nutritionist was responsible for the guidance of nutrition knowledge.

##### Training of the intervention team members

First, a training plan was developed, and the intervention group members received continuous training, including expertise, daily healthcare, lifestyle, diet, exercise and medication-related knowledge, with a focus on training in nutrition knowledge. The training was given once every month for 1–2 h. The training comprised the methods of group lecture, group discussion, on-site demonstration, group teaching and the distribution of the paper version of auxiliary materials. The intervention period was set at 3 months, 6 months and 1 year.

##### Implementation steps of the intervention programme

1. Develop personalised intervention guidance. On the day of discharge, the interventionists conduct a comprehensive evaluation of the patient, and after communicating with the doctors in the intervention team, they develop a personalised intervention plan and provide one-to-one health education and discharge guidance. The intervention period is 30 min.
2. On the day of discharge, patients and family caregivers are invited to join the WeChat group to facilitate the reception and exchange of information and better implementation of continuous care. The intervention plan is performed daily, weekly and monthly, and the intervention team members publish daily articles on knowledge about DN on the large medical kidney work platform. Telephone follow-up visits are performed at least every 2 weeks. Patients are invited to attend the health knowledge lecture hall every month, and family care groups are established. Nurses and nutritionists give patients and family caregivers comprehensive home care guidance and feedback patient information at any time.

#### Observation indicators

##### General patient sociological data

The general sociological data of the patients were obtained via questionnaire, including age, gender, educational level, marital status, residence type, income status and medical insurance type, and the file information of the patients was established.

##### Clinical evaluation indicators

The questionnaire score was used to investigate compliance behaviour before the intervention and at 3 and 6 months after the intervention. Quality of life was assessed using the quality-of-life assessment scale (SF-36). The improvement of the patients’ mastery of DN clinical knowledge and self-management efficacy before and after the intervention was also assessed, in addition to changes in the biochemical indicators of renal function before and after the intervention, mainly including urinary microalbumin/creatinine ratio (UACR), blood glucose level, blood urea nitrogen (BUN) and serum creatinine (sCr) level.

##### Data collection

Data collection and collation were completed before and after the intervention by the intervention nurse.

#### Statistical analysis method

We used the formula *n* = *Z*2 × SD2/*d*2 to calculate the required sample size for each group, where *n* is the sample size, *Z* is the *Z*-score corresponding to the confidence level, SD is the standard deviation, and *d* is the effect size. We assumed that *Z* = 1.96 (corresponding to a 95% confidence level), SD = 0.5 and *d* = 0.2 (corresponding to a small to medium effect). Based on these parameters, we calculated that the required sample size for each group was *n* = 24. To account for possible attrition or missing data, we increased the sample size for each group and finally determined that 40 patients were needed for each group. Statistical analysis was performed using SPSS22.0 software. Qualitative data were described in terms of number of cases and percentage for the *χ*^2^ test. Quantitative data were expressed as mean ± standard deviation for an unpaired *t* test. A *P* value of < 0.05 was considered to indicate a significant difference.

## Results

### Comparison of general data between the two groups

In our study, we compared the sociodemographic data of 80 participants, evenly split between the observation and control groups, as detailed in Table 1. The distribution of age, gender, education level, marital status, payment method, monthly household income and habitation showed no significant differences between the groups, indicating a well-balanced demographic profile at baseline among the study subjects.

**Table 1:** Sociodemographic data of the study subjects at baseline

Item	The observation group (*n* = 40)	The control group (*n* = 40)	*X* value	*P* value
Age				
40–49 years old	4	3	1.289	0.884
50–59 years old	12	15		
> 60 years old	24	22		
Gender				
Male	28	26	0.232	0.812
Female	12	14		
Education				
Junior high school and below	16	19	0.459	0.831
Senior high school	17	15		
University	7	6		
Marriage				
Unmarried	3	2	0.868	0.762
Married	32	35		
Bereft of one's spouse	3	2		
Divorced	2	1		
Mode of payment				
Medical insurance	38	39	0.721	0.675
At one’s own expense	2	1		
Per capita monthly household income				
< 1000	4	3	1.065	0.791
1000–3000	11	14		
3000–5000	15	16		
> 5000	10	7		
Habitation				
Rural area	11	10	0.621	0.733
Town	7	11		
City	22	19		

### Comparison of postintervention compliance behaviour between the two groups

The results showed that the total compliance scores of both groups reached the standard before the intervention. There was no statistically significant difference. At 3 and 6 months after the intervention, the compliance score decreased in both groups to varying degrees, but it was significantly higher in the observation group than in the control group. The differences were statistically significant (Tables 2, 3, 4).

**Table 2:** Comparison of preintervention compliance behaviours between the two groups

Group	Number	Correct medication	Reasonable diet	Exercise	Quit smoking and drinking	Regular return visit
The observation group	40	2.31 ± 0.53	2.47 ± 0.40	2.46 ± 0.38	2.42 ± 0.40	2.43 ± 0.43
The control group	40	2.28 ± 0.49	2.34 ± 0.50	2.26 ± 0.54	2.36 ± 0.50	2.29 ± 0.49
*T* value		− 0.263	− 1.284	-1.916	− 0.593	− 1.358
*P* value		0.793	0.203	0.059	0.555	0.178

**Table 3:** Comparison of compliance behaviour at 3 months after the intervention in both groups

Group	Number	Correct medication	Reasonable diet	Exercise	Quit smoking and drinking	Regular return visit
The observation group	40	2.11 + 0.52	2.27 + 0.38	1.98 + 0.39	2.02 + 0.41	2.25 + 0.42
The control group	40	1.88 + 0.51	1.98 + 0.50	1.67 + 0.54	1.78 + 0.50	1.87 + 0.49
*T* value		− 1.997	− 2.921	− 2.943	− 2.347	− 3.724
*P* value		0.049	0.005	0.004	0.02	0

**Table 4:** Comparison of compliance behaviour at 6 months after the intervention in both groups

Group	Number	Correct medication	Reasonable diet	Exercise	Quit smoking and drinking	Regular return visit
The observation group	40	2.08 + 0.64	2.12 + 0.51	1.87 + 0.50	2.23 + 0.49	2.17 + 0.60
The control group	40	1.53 + 0.60	1.65 + 0.69	1.44 + 0.61	1.72 + 0.76	1.81 + 0.51
*T* value		− 3.965	− 3.464	− 3.448	− 3.567	− 2.891
*P* value		0	0.001	0.001	0.001	0.005

### Comparison of patients’ mastery of disease knowledge and self-management ability

The results showed that the knowledge level increased in the observation group compared with the control group, indicating that the HCH linkage management mode changed the patients’ cognitive concept of the disease and increased the attention to DN. Changing the bad behaviour of patients plays a positive role in delaying the progression of the disease (Table 5).

**Table 5:** Comparison of mastery of disease knowledge and self-management ability

Group	The mastery of DN clinical knowledge (before the intervention)	The mastery of DN clinical knowledge (after the intervention)	Self-management (before the intervention)	Self-management (after the intervention)
The observation group	54.27 + 8.52	89.64 + 6.52	45.61 + 7.22	78.23 + 6.13
The control group	55.10 + 8.35	72.10 + 9.26	47.26 + 7.93	62.88 + 6.50
*T*	− 0.44	8.334	0.973	10.866
*P*	0.661	0	0.334	0

### Changes in biochemical indicators of renal function and blood glucose before and after intervention

The results indicated that after the intervention, the blood glucose level of the observation group was lower than that of the control group, as was the case with the sCr, BUN and UACR levels, suggesting that the intervention could effectively improve various renal function indicators and the blood glucose level of patients. This may be related to the closer communication between the patient and the doctor, with the intervention team members able to grasp the dynamic information of the patient’s condition change as soon as possible and adjust the treatment plan at any time. Details are shown in Tables 6 and 7.

**Table 6:** Changes in biochemical indicators of renal function in the two groups

Group	Clinical indicators	The observation group (*N* = 40)	The control group (*N* = 40)	*T* value	*P* value
Before the intervention	Serum creatinine	159.35 ± 19.75	156.66 ± 17.54	− 0.644	0.521
	Blood urea nitrogen	17.18 ± 5.16	16.35 ± 4.76	− 0.748	0.457
	Microprotein	217.31 ± 2.12	216.35 ± 2.46	− 1.87	0.065
After the intervention	Serum creatinine	126.39 ± 18.25	135.47 ± 18.36	2.218	0.029
	Blood urea nitrogen	9.32 ± 3.24	11.42 ± 3.76	2.676	0.009
	Microprotein	117.00 ± 2.31	162.32 ± 2.14	91.204	0.008

**Table 7:** Blood glucose of the patients before and after the intervention

Group	Blood glucose (before the intervention)	Blood glucose (after the intervention)	PBG (before the intervention)	PBG (after the intervention)
The control group	8.63 + 0.46	7.82 + 0.16	13.21 + 1.25	11.66 + 1.95
The observation group	8.59 + 0.42	7.01 + 025	13.32 + 1.14	9.92 + 1.63
*T* value	0.406	14.203	− 0.411	4.33
*P* value	0.686	0	0.682	0.001

## Discussion

The use of the HCH management model for DN is both necessary and important. Diabetic nephropathy is a common microvascular complication of diabetes mellitus and has become one of the main causes of death in the development of clinical end-stage renal disease [9, 10]. In the past, the routine management mode was adopted for diabetes and related complications, such as diabetes specialty management and self-management modes [11, 12]. Currently, the HCH linkage management mode is required alongside routine care, so that the scope of the management is gradually expanded, and the active cooperation and intervention of the family and society is crucial. The rich management content and the full inclusion of nutrients ensure that patients realise the importance of the self-management of diabetes. Reasonable nutrition management [13–15] and regular health lectures can effectively help control and delay the development of the disease and improve the quality of life of patients.

The HCH linkage management mode can improve patients’ compliance behaviour, cognition of the disease and their self-management efficiency. The results of this study showed that the total compliance scores of both groups reached the standard before the intervention. At 3 and 6 months after the intervention, the compliance behaviour score decreased in both groups but was significantly higher in the observation group than in the control group. This may be related to the decreased attention to the disease of the patients and their families and the lack of return visits on time. However, this shows that the HCH linkage management mode is a diversified health education and continuous care mode, encouraging patients to develop good medical compliance behaviour. The study results showed that the disease knowledge score was significantly higher in the observation group after the intervention compared with that of the control group, indicating that the HCH linkage management mode changed the cognitive concept of the patients and increased their attention to DN. Through the guidance on health behaviour, the personalised nutrition guidance and the development of the health lectures in the observation group, the patients could learn more about DN, further strengthening their knowledge of DN prevention, treatment and nursing. Changing the bad behaviour of patients plays a positive role in delaying the progression of the disease [16, 17]. Studies have shown that among patients with chronic kidney disease, they do not adhere to the recommended diet well after receiving dietary guidance from a dietitian. Dietary management depends more on the willpower of the patients themselves, which requires the nutrition education staff to strengthen education and diet management, as well as choose the appropriate nutrition management mode [18].

In this study, we found that the HCH linkage management model could improve renal function indicators in patients with DN. The results showed that after the intervention, the blood glucose level of the observation group was lower than that of the control group, as was the case with the sCr, BUN and UAER levels, suggesting that the intervention could effectively improve various renal function indicators and the blood glucose level of patients. This may be related to the closer communication between the patient and the doctor, with the intervention team members able to grasp the dynamic information related to the patient’s change in condition as soon as possible and adjust the treatment plan at any time. This further formally confirms the feasibility and effectiveness of the HCH linkage management model.

Recent studies have shown that a new nutrition management model of ‘remote personalised management based on Internet + diet’ has a good nutritional treatment effect in patients with DN. One study found that after the intervention, the blood Cr and UACR levels increased in the two groups, whereas the estimated GFR (eGFR), fasting blood glucose and glycated haemoglobin levels decreased compared with before the intervention, and the differences in blood Cr, UACR and eGFR before after the intervention were significant in the control group [19]. This is similar to our findings. The choice of appropriate nutrition management mode is conducive to the improvement of renal function in patients with DN and confirms the necessity of the related research.

In conclusion, the purpose of HCH linkage management mode is not only to treat diseases but also to control and prevent them. In this study, a DN nutritional management team was established, and the information of patients and the self-management ability of discharged patients were made available at any time through a WeChat group. In addition, health education regarding relevant knowledge was conducted to ensure that the management of patients with DN could be effective in real time. The implementation of the HCH linkage management mode for patients with DN improves their mastery of disease knowledge and self-management efficiency, enhances the compliance behaviour and the quality of life of patients, and delays the progress of the disease.

## Data Availability

The datasets used and analysed during the current study are available from the corresponding author on reasonable request.
